# Reconceptualizing family stigma in the context of mental illness

**DOI:** 10.1192/j.eurpsy.2026.10911

**Published:** 2026-07-31

**Authors:** C. C. Williams

**Affiliations:** 1 Factor-Inwentash Faculty of Social Work; 2 Temerty Faculty of Medicine, Rehabilitation Sciences Institute, University of Toronto, Toronto, Canada

## Abstract

**Introduction:**

Stigma related to mental illness has been studied primarily in relation to individuals diagnosed with illness. Families, however, encounter stigma in unique and complex ways. Classic definitions of stigma locate it at the intersection of stereotypes, prejudice, and discrimination—processes extensively theorized regarding individuals with mental illness but less so for their families. Further, much of the literature conflates “family burden” with stigma and under-theorizes the distinct mechanisms shaping family experiences. Consequently, family psychoeducation and support services do not address the layered stigma families face.

**Objectives:**

This paper reconceptualizes family stigma through an expanded framework that moves beyond the prevailing focus on “associative stigma” to consider how the whole family, including diagnosed individuals, is affected by stigma enacted in interpersonal, cultural, and structural processes.

**Methods:**

The analysis draws on a literature review and qualitative data from focus groups in Ontario, Canada, involving participants who identified in one or more roles as service providers, family members, and people diagnosed with mental illness. Audio recordings of the focus groups were transcribed verbatim and analyzed using template analysis, which allowed for a flexible yet structured approach to identifying themes.

**Results:**

Three interrelated dimensions of family stigma emerged:

**Associative stigma:** negative judgments transferred to family members by association.

**Intimate stigma:** enacted within family relationships, influencing secrecy, belonging, and caregiving.

**Structural stigma:** embedded in policies, institutional practices, and systemic inequities affecting family resources and supports.

This framework clarifies how stigma affects families, constrains caring relationships, and restricts their pursuit of family wellbeing.

**Conclusions:**

Family stigma is not reducible to “burden” but is a multi-level phenomenon manifesting through associative judgments, intimate dynamics, and structural inequities. By situating family experiences within relational and systemic contexts, this reconceptualization enriches stigma theory and underscores the value of family-centered, culturally responsive, and intersectional approaches. For practice and policy, it calls for explicit integration of anti-stigma content in psychoeducational programming and services to empower family members to support each other in challenging stigmatizing discourses that undermine their wellbeing.

**Disclosure of Interest:**

None Declared

